# Triplet Excited Carbonyls and Singlet Oxygen Formation During Oxidative Radical Reaction in Skin

**DOI:** 10.3389/fphys.2018.01109

**Published:** 2018-08-15

**Authors:** Ankush Prasad, Anastasiia Balukova, Pavel Pospíšil

**Affiliations:** Department of Biophysics, Centre of the Region Haná for Biotechnological and Agricultural Research, Faculty of Science, Palacký University, Olomouc, Czechia

**Keywords:** singlet oxygen, triplet excited carbonyl, ultra-weak photon emission, two-dimensional photon imaging, skin

## Abstract

The skin is the largest organ in the body and is consistently exposed to aggressive environmental attacks (biological/physical/chemical, etc.). Reactive oxygen species (ROS) are formed during the normal oxidative metabolism which enhances to a lethal level under stress conditions referred to as oxidative stress. While, under normal conditions, cells are capable of dealing with ROS using non-enzymatic and enzymatic defense system, it can lead to a critical damage to cell system via the oxidation of cellular components under stress condition. Lipid peroxidation is a well-established mechanism of cellular injury in all kinds of organisms and it is often used as an indicator of oxidative stress in cells and tissues. In the presence of metal ions, ROS such as hydrogen peroxide (H_2_O_2_) produces highly reactive hydroxyl radical (HO^•^) via Fenton reaction. In the current study, we have used the porcine skin (intact pig ear/skin biopsies) as an *ex vivo*/*in vitro* model system to represent human skin. Experimental results have been presented on the participation of HO^•^ in the initiation of lipid peroxidation and thereby leading to the formation of reactive intermediates and the formation of electronically excited species eventually leading to ultra-weak photon emission (UPE). To understand the participation of different electronically excited species in the overall UPE, the effect of a scavenger of singlet oxygen (^1^O_2_) on photon emission in the visible and near-infrared region of the spectrum was measured which showed its contribution. In addition, measurement with interference filter with a transmission in the range of 340–540 nm reflected a substantial contribution of triplet carbonyls (^3^L=O^∗^) in the photon emission. Thus, it is concluded that during the oxidative radical reactions, the UPE is contributed by the formation of both ^3^L=O^∗^ and ^1^O_2_. The method used in the current study is claimed to be a potential tool for non-invasive determination of the physiological and pathological state of human skin in dermatological research.

## Introduction

**FIGURE 1:**
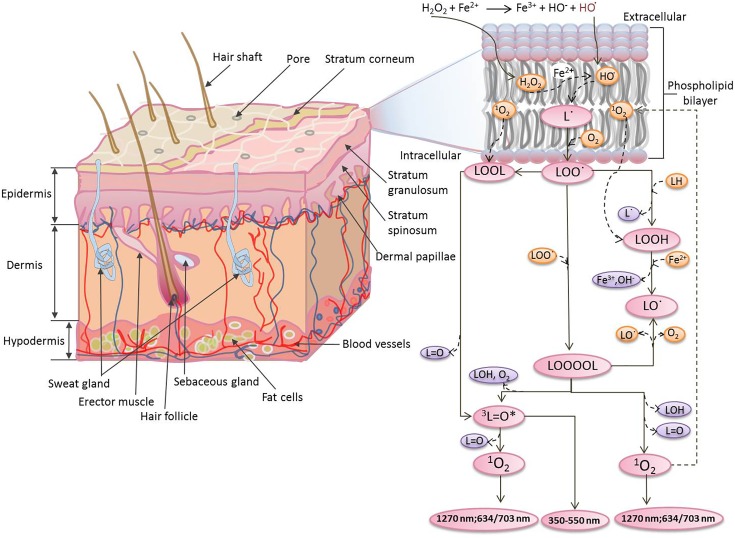
Mechanism of the formation of electronically excited species by oxidative metabolic processes via oxidation of polyunsaturated fatty acid initiated by Fenton’s reagent. The Fenton’s reagent generates hydroxyl radical (HO^•^) at different locations within the vicinity of phospholipid bilayer.

The skin plays diverse essential functions such as protection against environment, metabolism, thermoregulation, sensation, and excretion (Zouboulis, 2009; Morrow and Lechler, 2015). The skin consists of the epidermis, which forms the outermost layer followed by dermis and subcutis/hypodermis which is the deepest layer (Meyer et al., 1994; Prost-Squarcioni, 2006;

Chartier et al., 2017). The whole epidermis constantly renews itself within few weeks and new cells are made in the lower layers of the epidermis (Rinnerthaler et al., 2015). The dermis contains extracellular molecules secreted by support cells that provide structural and biochemical support to the adjacent/surrounding cells and also consists of a dense network of tough elastic collagen fibres and bundles of proteins (elastin) found in the extracellular matrix. These make the skin strong and robust while at the same time elastic (Tepole et al., 2012). The subcutis/hypodermis is mostly made up of fat and connective tissue. In the subcutis, there are tiny cavities which are filled with storage tissue made out of fat and water (**Figure 1**). During the oxidative stress generated by abiotic stresses (toxic chemicals, exposure to ultraviolet irradiations, etc.), the epidermal and dermal cells are known to be most affected (Rinnerthaler et al., 2015; Ji and Li, 2016).

Apart from ethical reasons, there are also methodological difficulties to work with human skins and thus are generally replaced by an animal model for *in vivo* experimental research (Hikima et al., 2012; Abdullahi et al., 2014). The selection of an animal model may depend on factors such as its availability, ease of handling and, most importantly, functional and anatomical similarity to that of humans. For larger scale testing of new agents/cosmetics etc., small mammals are frequently used; however, these animals differ from humans in important anatomical and physiological ways (Kong and Bhargava, 2011). Based on the above consideration, porcine skin is considered to be the most appropriate model, from the perspective of dermatological investigation among all other experimental models. Several studies have demonstrated that porcine skin has important similarities in morphology, composition, and immunoreactivity to that of human skin (Avon and Wood, 2005). Porcine skin has thicker epidermis which is a striking similarity with human skin (Jacobi et al., 2007). The epidermis of the pig is reported to vary in thickness from 30 to 140 μm, thus being within a range similar to human skin which is in the range of 10 to 120 μm (Meyer et al., 1978; Morris and Hopewell, 1990; Avon and Wood, 2005).

During the last few decades, ultra-weak photon emission (UPE) detection techniques have been extensively used to study the oxidative metabolic processes in the different living system *in vivo*, *ex vivo*, and *in vitro* (Kobayashi, 2005; Cifra and Pospíšil, 2014; Ou-Yang, 2014; Poplova et al., 2017). Keratinocytes, fibroblast, skin homogenate, *ex vivo* skin tissues as well as malignant skin cells have been measured *in vitro* (Torinuki and Miura, 1981; Niggli et al., 2008; Madl et al., 2017). Spontaneous and induced UPE under exposure to stress factors such as ultra-violet irradiations, smoke and toxic chemicals have also been documented for human skin/animals cells model/organism and have been well-summarized in recent reviews (Sauermann et al., 1999; Ou-Yang, 2014).

Reactive oxygen species (ROS) has been reported to contribute to UPE via oxidation of biomolecules such as lipids, proteins and nucleic acids (Prasad and Pospíšil, 2011a,b; Rastogi and Pospíšil, 2011; Poplova et al., 2017). In the current study, we have used porcine ear and skin biopsies as a model system to represent human skin. We have measured the spontaneous and induced UPE from the porcine ear (as an *ex vivo* model system) and skin biopsies (as an *in vitro* model system). The induced UPE was measured under the exogenous application of Fenton’s reagent generated chemically and applied topically on the skin before the start of photon emission measurement. Clinically, iron released by hemoglobin may initiate free radical chain reactions and may lead to ROS overproduction followed by lipid peroxidation (Sadrzadeh et al., 1984; Rifkind et al., 2015). As a result of iron-induced Fenton reaction, hydroxyl radical (HO^•^) is known to be produced, which is known to be among highly reactive and short-lived species. The iron in the free form favors the conversion of lipid hydroperoxides (LOOH) to lipid alkoxyl (LO^•^) radicals (**Figure 1**). The electronically excited species generated as a product of the oxidative radical reaction were investigated and their participation in the UPE has been presented.

## Materials and Methods

### Porcine Skin

Intact pig ears were collected from a local slaughter house and transported at low temperature within first 30 min. Skin biopsies were prepared as per the procedure described with minor modifications (Chiu and Burd, 2005). For each set of measurements, fresh skin samples collected each day were used.

### Chemicals

Fenton’s reagent was prepared using hydrogen peroxide (H_2_O_2_) (Sigma-Aldrich Chemie GmbH, Germany) and ferrous sulfate (FeSO_4_.7H_2_O) (BDH Laboratory Supplies, United Kingdom). A fixed concentration of FeSO_4_ (500 μM) and a variable concentration of H_2_O_2_ (100 μM/1 mM) was used_._ Spin trapping reagent, POBN [α-(4-Pyridyl 1-oxide)-*N*-tert-butylnitrone] was purchased (Sigma-Aldrich Chemie GmbH, Germany).

### EPR Spin-Trapping Spectroscopy

**FIGURE 2:**
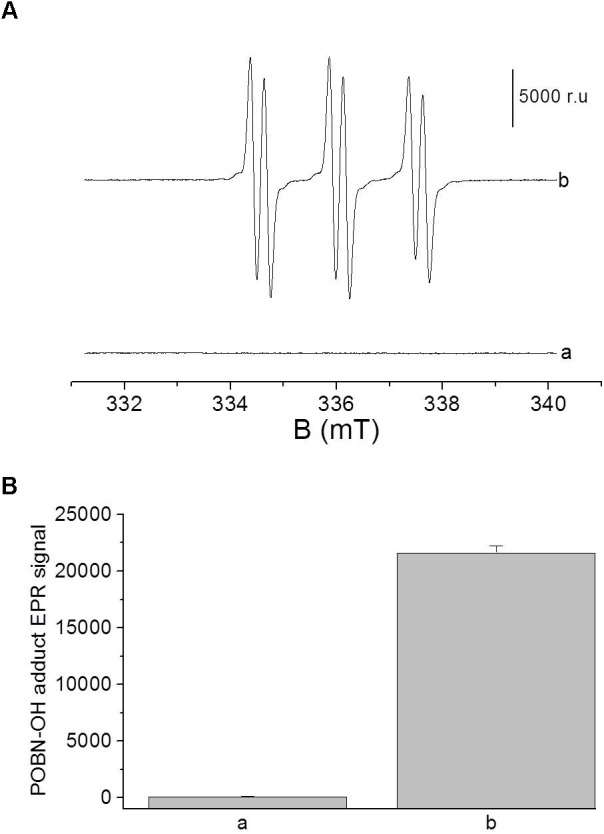
**(A)** Electron paramagnetic resonance (EPR) spectra of POBN-OH adduct was detected in solutions containing 100 μM FeSO_4_ in the absence (a) and presence of 20 μM (b) of H_2_O_2_. Bar represent 5000 relative units. **(B)** The data are presented as the mean and standard deviation of three measurements.

To confirm the formation of HO^•^ during the Fenton reaction, electron paramagnetic resonance (EPR) spectra of POBN (4-pyridyl-1-oxide-*N*-tert-butylnitrone)-OH adduct was detected at 20 μM H_2_O_2_ in the presence of 100 μM FeSO_4_ (**Figure 2**). Hydroxyl radical was detected using POBN [25 mM] utilizing spin-trapping in a glass capillary tube (Blaubrand intraMARK, Brand, Germany). EPR spectra were recorded using an EPR spectrometer MiniScope MS400 (Magnettech GmbH, Berlin, Germany) with following EPR conditions: microwave power, 10 mW; modulation amplitude, 1 G; modulation frequency, 100 kHz; sweep width, 100 G; scan rate, 1.62 G s^-1^, gain, 100.

### Measurement Setup and Experimental Conditions

**FIGURE 3:**
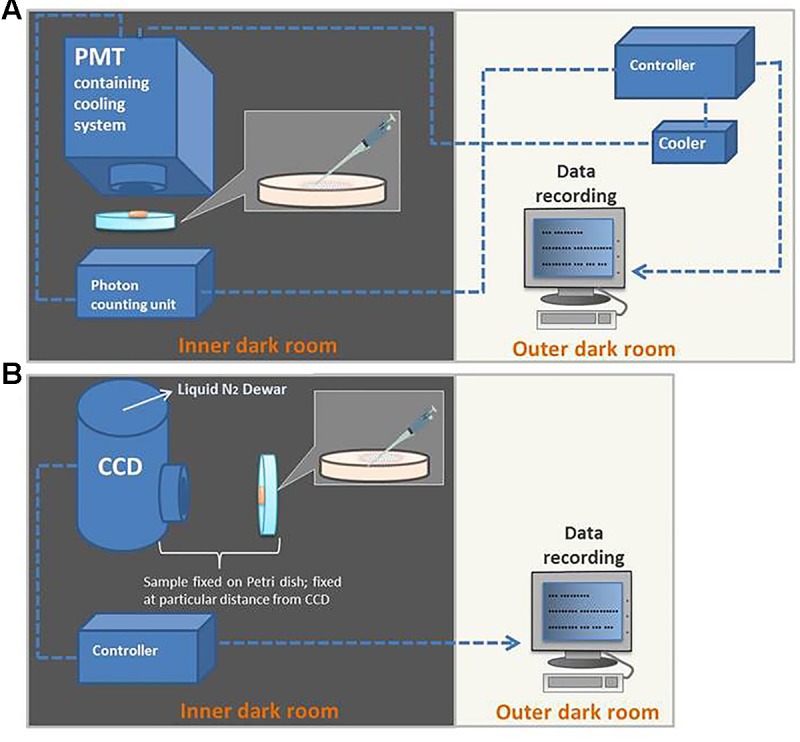
Schematic illustration of the experimental setup for detection of kinetics of ultra-weak photon emission (UPE) using PMT **(A)** and two-dimensional imaging of UPE using CCD camera **(B)**.

It is a pre-requisite to specifically design a dark room to avoid any kind of interference from stray photons. In the current study, all UPE measurements were performed in an experimental dark room as described in Prasad and Pospíšil (2013). A schematic dark room and measurement setup are shown in **Figure 3**. All experiments were done in three replicates and the representative graph has been presented.

#### Fenton’s Reagent-Induced Kinetic Measurement of Ultra-Weak Photon Emission From Skin

**FIGURE 4:**
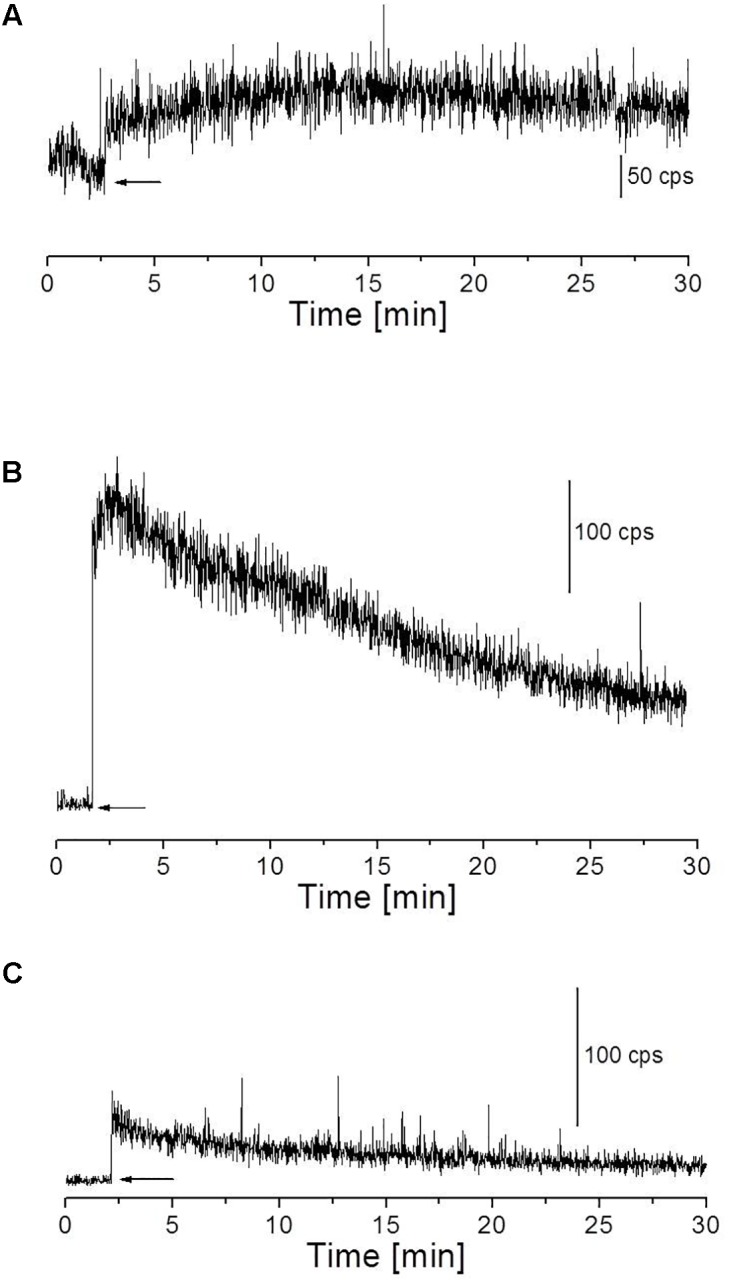
Fenton’s reagent-induced UPE measured using visible PMT from the porcine skin sample. **(A)** Kinetics of UPE was measured after the topical application of Fenton’s reagent (100 μM H_2_O_2_ containing 500 μM FeSO_4_). **(B)** Kinetics of UPE was measured after the topical application of Fenton’s reagent (1 mM H_2_O_2_ containing 500 μM FeSO_4_). **(C)** Kinetics of UPE was measured in the presence of sodium ascorbate (5 mM) applied to the skin prior to topical application of Fenton’s reagent (1 mM H_2_O_2_ containing 500 μM FeSO_4_). The decay curve was measured for a duration of 30 min. The arrow indicates the application of chemicals.

The skin biopsies were subjected to topical application of Fenton’s reagent in the concentration of 500 μM FeSO_4_ and 100 μM H_2_O_2_ (**Figure 4A**) or 1 mM H_2_O_2_ (**Figure 4B**). These concentrations of Fenton’s reagent were chosen based on the pilot experiments in which the effect of different concentrations on photon emission was extensively explored (**Supplementary Figures S1**, **S2**). Fenton’s reagent was always topically applied after the start of measurements (indicated by arrows). When required, 5 mM sodium ascorbate (Sigma-Aldrich Chemie GmbH, Germany) was added 20 s prior to the topical application of Fenton’s reagent.

#### Fenton’s Reagent-Induced Ultra-Weak Photon Emission in the Blue–Green Region of the Spectrum

**FIGURE 5:**
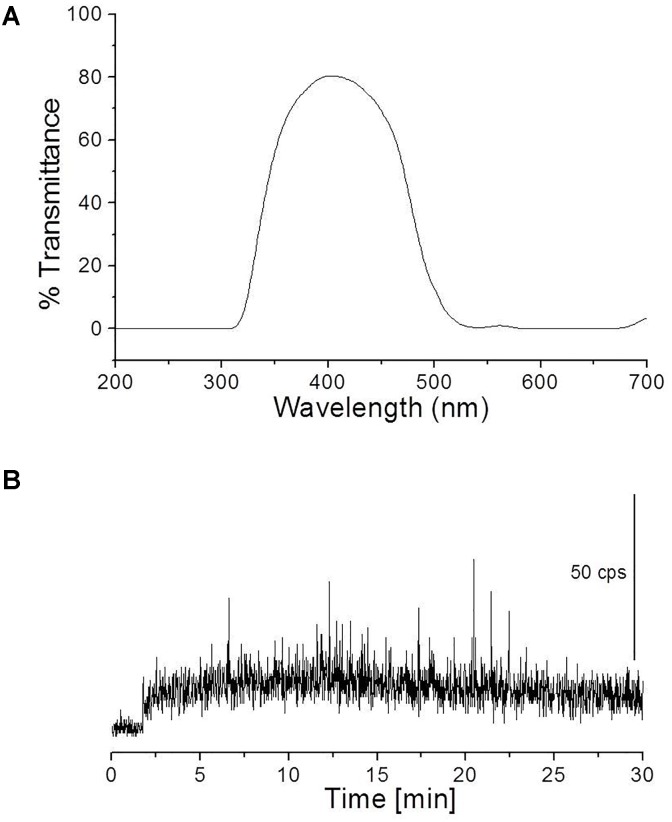
Transmission spectrum of interference filter type 644 (Schott & Gen., Jena, Germany) **(A)** and kinetics of UPE measured after the topical application of Fenton’s reagent (containing 1 mM H_2_O_2_ and 500 μM FeSO_4_) in the presence of interference filter type 644 (340–540 nm) **(B)**. Other experimental conditions as described in **Figure 4**.

To study the spectral distribution of ultra-weak photons emitted during the oxidative radical process mediated by Fenton’s reagent, a blue–green interference filter type 644 (Schott & Gen., Jena, Germany) with a transmission in the range 340–540 nm was mounted in front of PMT window (**Figure 5A**). The kinetics of UPE was measured from the porcine skin biopsies after the topical application of Fenton’s reagent (1 mM H_2_O_2_ containing 500 μM FeSO_4_).

### Ultra-Weak Photon Emission

#### Two-Dimensional Imaging of Ultra-Weak Photon Emission

Two-dimensional imaging of UPE was measured in porcine ear/skin biopsies using highly sensitive CCD camera. All samples were dark-adapted for 30 min to eliminate any interference by delayed luminescence. Other conditions are as per the procedure described in listed references (Prasad and Pospíšil, 2011b; Prasad et al., 2016). CCD camera VersArray 1300B (Princeton Instruments, Trenton, NJ, United States) with the spectral sensitivity of 350–1000 nm and almost 90% quantum efficiency in the visible range of the spectrum was used under following parameters: scan rate, 100 kHz; gain, 2; an accumulation time, 30 min/45 min (porcine ear/skin biopsies). CCD camera was cooled down to -104°C using a liquid-nitrogen cooling system for reduction of dark current. Before each measurement, the data correction was made by subtracting the background noise.

#### Kinetics of Ultra-Weak Photon Emission in the Visible Region and Near-Infrared Region of the Spectrum

The kinetics of photon emission in the visible region was performed using PMT R7518P (spectral sensitivity: 185–730 nm; detection area: Ø 28 mm). PMT was cooled down to -30°C using thermoelectric cooler C9143 (Hamamatsu Photonics K.K., Iwata City, Japan) for reduction of thermal electrons. UPE in the near-infrared region was measured using a high-speed near-infrared PMT H10330C-45 (Hamamatsu Photonics K.K., Iwata City, Japan) (spectral sensitivity: 950–1400 nm; detection area: Ø 18 mm). The measurements were performed at room temperature. The photon counts were recorded using low-noise photon counting unit (C9744, Hamamatsu Photonics K.K., Iwata City, Japan).

## Results

### Fenton’s Reagent-Induced Kinetic Measurement of Ultra-Weak Photon Emission From Skin

The kinetics of UPE was measured from the porcine skin biopsies after the topical application of Fenton’s reagent using visible PMT (**Figures 4A,B**). Prior to measurements, the dark count in the experimental dark room was measured and recorded to be ∼2 counts s^-1^ (**Supplementary Figure S3A**). As additional controls, the photon emission from H_2_O_2_, Fenton’s reagent and scavenger were measured separately/in combinations to test any kind of interference/contribution in overall UPE. It was found that the contribution of chemicals (in the absence of skin sample) showed signal intensity corresponding to the photon count as observed in dark (**Supplementary Figures S3B–E**). When skin biopsies were subjected to topical application of Fenton’s reagent in the concentration of 500 μM FeSO_4_ and 100 μM H_2_O_2_ (**Figure 4A**) or 1 mM H_2_O_2_ (**Figure 4B**), it can be observed that the UPE was enhanced to ∼80 counts s^-1^ and ∼250 counts s^-1^ under exogenous application of lower and higher concentrations of Fenton’s reagent, respectively which then decayed over time. Based on the current observation, it can be concluded that the concentration of Fenton’s reagent which acts as an oxidant for the biomolecules (described in the later section) contributes as a key factor for ROS-mediated UPE. For all other results presented in the next section, we have chosen 1 mM H_2_O_2_ containing 500 μM FeSO_4_ as the inducer of UPE (as otherwise indicated).

### Fenton’s Reagent-Induced Ultra-Weak Photon Emission in the Blue–Green Region of the Spectrum

To study the spectral distribution of ultra-weak photons emitted during the oxidative radical process mediated by Fenton’s reagent, we mounted an interference filter (type 644 with a transmission in the range 340–540 nm) (**Figure 5A**) in the front of PMT window. Kinetics of UPE was measured from the porcine skin biopsies after the topical application of Fenton’s reagent (1 mM H_2_O_2_ containing 500 μM FeSO_4_). It can be observed that the application of Fenton’s reagent resulted in UPE of ∼20 counts s^-1^ in contrary to 250 counts s^-1^ without interference filter (**Figures 4B**, **5B**). The current observation clearly indicates that not all UPE observed in Fenton’s reagent-induced process is contributed by species emitting in the blue–green region of the spectrum but can be due to the involvement of other electronically excited species. A small decrease in photon emission; however, can be also contributed by % transmittance of the interference filter.

### Fenton’s Reagent-Induced Imaging of Ultra-Weak Photon Emission From Skin

**FIGURE 6:**
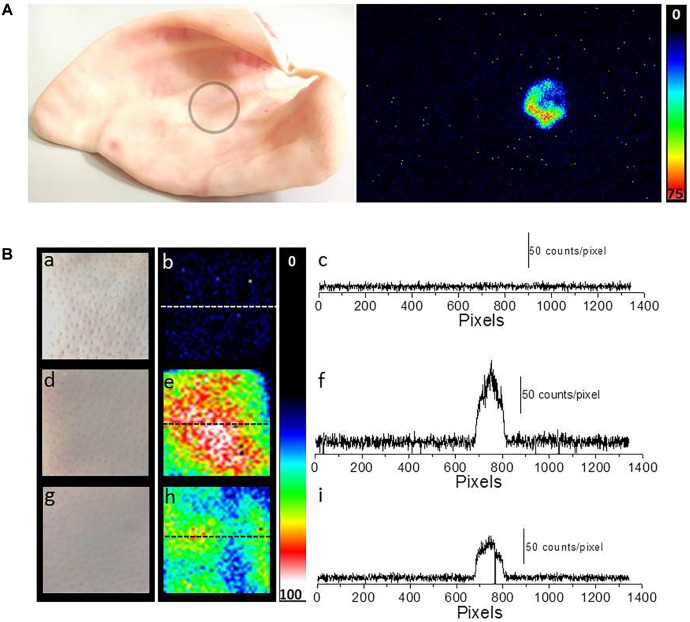
Two-dimensional Fenton’s reagent-induced UPE measured using CCD camera from the porcine ear/skin biopsies. **(A)** Photograph of pig ear (circle represents the area of the porcine ear where Fenton’s reagent was topically applied) and two-dimensional UPE imaging measured after the topical application of Fenton’s reagent (1 mM H_2_O_2_ containing 500 μM FeSO_4_). **(B)** Photographs (a, d, and g) and corresponding images of UPE of spontaneous (b), induced with Fenton’s reagent (1 mM H_2_O_2_ containing 500 μM FeSO_4_) (e) and induced with Fenton’s reagent (1 mM H_2_O_2_ containing 500 μM FeSO_4_) in the presence of sodium ascorbate (5 mM) (h). Figure **(B)** (c, f, and i) shows the spatial profile of the photon emission in a single strip of the image (dashed line) in spontaneous (c), Fenton’s reagent-induced (f) and Fenton’s reagent-induced in the presence of sodium ascorbate (i). Y-axis reflects the number of photon counts accumulated after 30 min, whereas the X-axis denotes the pixel of the image.

Two-dimensional UPE imaging was measured from the porcine ear/skin biopsies after the topical application of Fenton’s reagent using CCD camera (**Figure 6**). **Figure 6A** shows the photograph (left panel) and imaging of UPE (right panel) from an *ex vivo* porcine ear. In **Figure 6A**, UPE imaging was performed after the treatment with Fenton’s reagent (1 mM H_2_O_2_) containing 500 μM FeSO_4_. **Figure 6B** shows the photograph (left panel; a, d, and g), imaging of UPE (middle panel; b, e, and h) and intensity of UPE (right panel; c, f, and i) from skin biopsies. The imaging of UPE was measured in the absence (b) and presence of Fenton’s reagent (e and h). In (e), Fenton’s reagent was applied to skin biopsy and measured subsequently while in (h); sodium ascorbate (5 mM) which is a well-known scavenger of singlet oxygen (^1^O_2_) was added prior to the topical application of Fenton’s reagent. It can be observed that the addition of sodium ascorbate prior to application of Fenton’s reagent significantly suppressed the UPE from the skin biopsy. As evident from the intensity of UPE, the skin untreated with Fenton’s reagent (c) does not show any increase while the skin treated with Fenton’s reagent (1 mM H_2_O_2_) containing 500 μM FeSO_4_ shows an intensity maximum of ∼150 counts/pixel which was found to be suppressed by ∼50% in the skin biopsy pre-treated with sodium ascorbate. Based on the current observation, it is evident that contribution of ^1^O_2_ dimol photon emission in the overall UPE observed cannot be completely ruled out. The current observation was further validated by measuring the effect of sodium ascorbate on Fenton’s reagent (1 mM H_2_O_2_) containing 500 μM FeSO_4_ on skin biopsy. It was observed that in the presence of sodium ascorbate, the UPE was suppressed by ∼5 times (**Figure 4C**).

### Fenton’s Reagent-Induced Ultra-Weak Photon Emission in the Near-Infrared Region of the Spectrum

**FIGURE 7:**
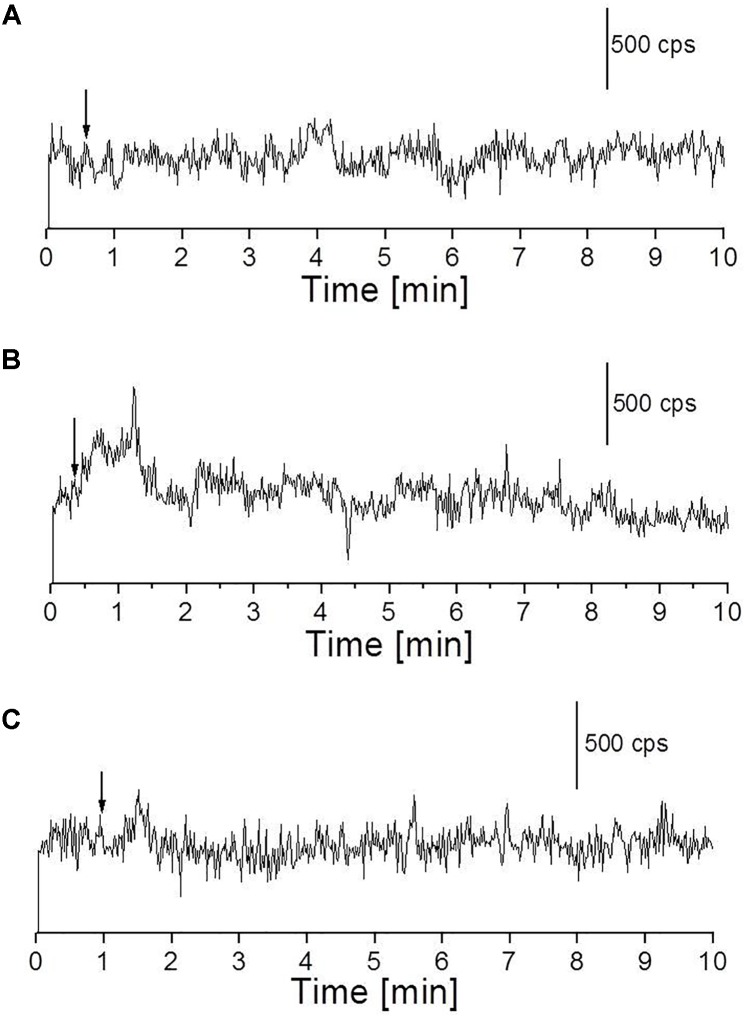
Fenton’s reagent-induced UPE measured using near-infrared PMT from the porcine skin sample. **(A)** Kinetics of UPE was measured after the topical application of Fenton’s reagent (100 μM containing 500 μM FeSO_4_). **(B,C)** Kinetics of UPE was measured after the topical application of Fenton’s reagent (1 mM H_2_O_2_ containing 500 μM FeSO_4_) added between 30 s and 1 min of the start of measurement (indicated by arrow) in the absence **(B)** and presence **(C)** of sodium ascorbate (5 mM), respectively added prior to treatment with Fenton’s reagent. The decay curve was measured for a duration of 10 min.

We measured the kinetics of UPE in the near-infrared region using a high-speed near-infrared PMT with a spectral sensitivity in the range of 950–1400 nm. The skin biopsy was subjected to topical application of Fenton’s reagent in the concentration of 100 μM H_2_O_2_ (A) and 1 mM H_2_O_2_ (B) containing 500 μM FeSO_4_. It can be observed that application of lower concentration (100 μM) of Fenton’s reagent did not enhance detectable range of UPE while application of higher concentration (1 mM) of Fenton’s reagent enhanced the UPE to about 500 counts s^-1^ which then decayed in the time range of 0–2 min (**Figures 7A,B**). Using near-infrared PMT, Fenton’s reagent-induced kinetics of UPE was measured subsequently in the presence of sodium ascorbate (**Figure 7C**). It can be observed that the addition of sodium ascorbate prior to application of Fenton’s reagent significantly suppressed the UPE as in agreement with results obtained in **Figure 6B** (h and i) and **Figure 4C**.

## Discussion

### Oxidative Radical Reaction and Triplet Excited Carbonyls in Overall Ultra-Weak Photon Emission

The oxidation of polyunsaturated fatty acid mediated by HO^•^ initiates with the hydrogen abstraction from the hydrophobic tail of the lipid molecule (L) resulting in the formation of an alkyl radical (L^•^), which in the presence of molecular oxygen (O_2_) forms lipid peroxyl radical (LOO^•^) (Halliwell and Gutteridge, 2007). The epidermal and the dermal layer of the porcine or human skin consists of a high distribution of this target and thus, access to the lipid molecules and further oxidation is very probable. With the formation of LOO^•^ and further accumulation, the interaction with another LOO^•^ becomes feasible. Self-reaction of LOO^•^ yields triplet carbonyls (^3^L=O^∗^) and O_2_ or the ground state of carbonyls (L=O) and ^1^O_2_ via the formation of tetroxide (LOOOOL) (**Figure 1**) (Russell, 1957; Cadenas and Sies, 2000; Miyamoto et al., 2014). In addition, LOO^•^ can react with neighboring lipid molecule and can lead to the formation of LOOH. Alternatively, cyclic high-energy intermediates dioxetanes (LOOL) can be formed by the cyclisation of LOO^•^ (Corey and Wang, 1994). As a result of oxidative metabolic processes, electronically excited species such as ^3^L=O^∗^ are formed by the decomposition of high-energy intermediates (LOOL and LOOOOL) (**Figure 1**) (Adam and Cilento, 1982; Cilento and Adam, 1995). The suppression of Fenton’s reagent-induced UPE from the porcine skin in the presence of blue–green filter was significant. It clearly indicates that ^3^L=O^∗^ is one of the major contributors in the overall UPE (**Figure 5**). The involvement of ^3^L=O^∗^ in UPE have been recently reported in several studies (Havaux, 2003; Footitt et al., 2016); however, the participation of other molecules cannot be completely ruled out. The decomposition of high-energy intermediates (LOOL and LOOOOL) leads to the formation of ^3^L=O^∗^ which can undergo an electronic transition from the triplet excited state to the ground state emitting ultra-weak photons in the near UVA and blue–green regions of the spectrum (350–550 nm).

### Oxidative Radical Reaction and Singlet Oxygen in Overall Ultra-Weak Photon Emission

In the presence of O_2_, the excitation energy can be transferred from ^3^L=O^∗^ to O_2_ via triplet-singlet energy transfer and can lead to the formation of ^1^O_2_ (Kellogg, 1969). The spontaneous collision of two ^1^O_2_ results in the dimol photon emission in the red region of the spectrum at the wavelengths of 634 and 703 nm or ^1^O_2_ can undergo from singlet excited state to ground triplet state accompanied by the monomol photon emission in the near IR region of the spectrum at the wavelengths of 1270 nm (Cadenas et al., 1980; Mathew and Roy, 1992; Miyamoto et al., 2007; Suzuki et al., 2008; Massari et al., 2011; Pospíšil et al., 2014). Our observation that UPE was significantly suppressed with the topical application of sodium ascorbate in Fenton’s reagent-induced UPE from porcine skin indicates that ^1^O_2_ can contribute either directly through dimol emission or indirectly can be involved in the formation of LOOL (**Figure 1**) to contribute to overall UPE. In agreement to this, two-dimensional imaging of Fenton’s reagent-induced UPE shows significant suppression in the presence of sodium ascorbate (**Figure 6B**). Our observation that UPE under the effect of Fenton’s reagent was enhanced in the near-infrared region of the spectrum and subsequently suppressed by the exogenous application of sodium ascorbate confirms the generation of ^1^O_2_ during the oxidative radical reaction.

## Conclusion

The current study presents the mechanism on the oxidation of polyunsaturated fatty acid which is one of the primary targets of ROS in the skin. It is aimed to clarify the participation of different electronically excited species (^3^L=O^∗^ and ^1^O_2_) in UPE during the oxidative radical reactions. The results presented by means of UPE kinetic measurement and two-dimensional imaging provides a series of evidence showing the contribution of these species in the overall UPE. The methodology used to obtain the information/results clearly indicates the potential of UPE as a non-invasive tool without the involvement of any probes, etc. The changes in UPE were observed to reflect the oxidative stress which can serve as a potential tool for monitoring the physiological and pathological state of a biological system. However, technical advancement with respect to sensitivity of PMT and CCD camera is essential for its wide application in different areas such as dermatological research and/or clinical applications.

## Author Contributions

AP and PP contributed to the conception and design of the work. AP analyzed, interpreted the data, and drafted the manuscript. AB participated in the drafting of the manuscript. PP revised it critically for important content. All authors approved the final version of the manuscript.

## Conflict of Interest Statement

The authors declare that the research was conducted in the absence of any commercial or financial relationships that could be construed as a potential conflict of interest. The reviewers SC and NE and handling Editor declared their shared affiliation at the time of the review.

## Abbreviations

CCD: charge-coupled device
PMT: photomultiplier tube
ROS: reactive oxygen species
