# Supplementary material for: Triplet Excited Carbonyls and Singlet Oxygen Formation During Oxidative Radical Reaction in Skin
Source: Front Physiol. 2018 Aug 15;9:1109. doi: 10.3389/fphys.2018.01109 (PMC6104306; doi:10.3389/fphys.2018.01109)
Supplement: Supplementary file 1 [file Presentation_1.pptx]

## Slide 1
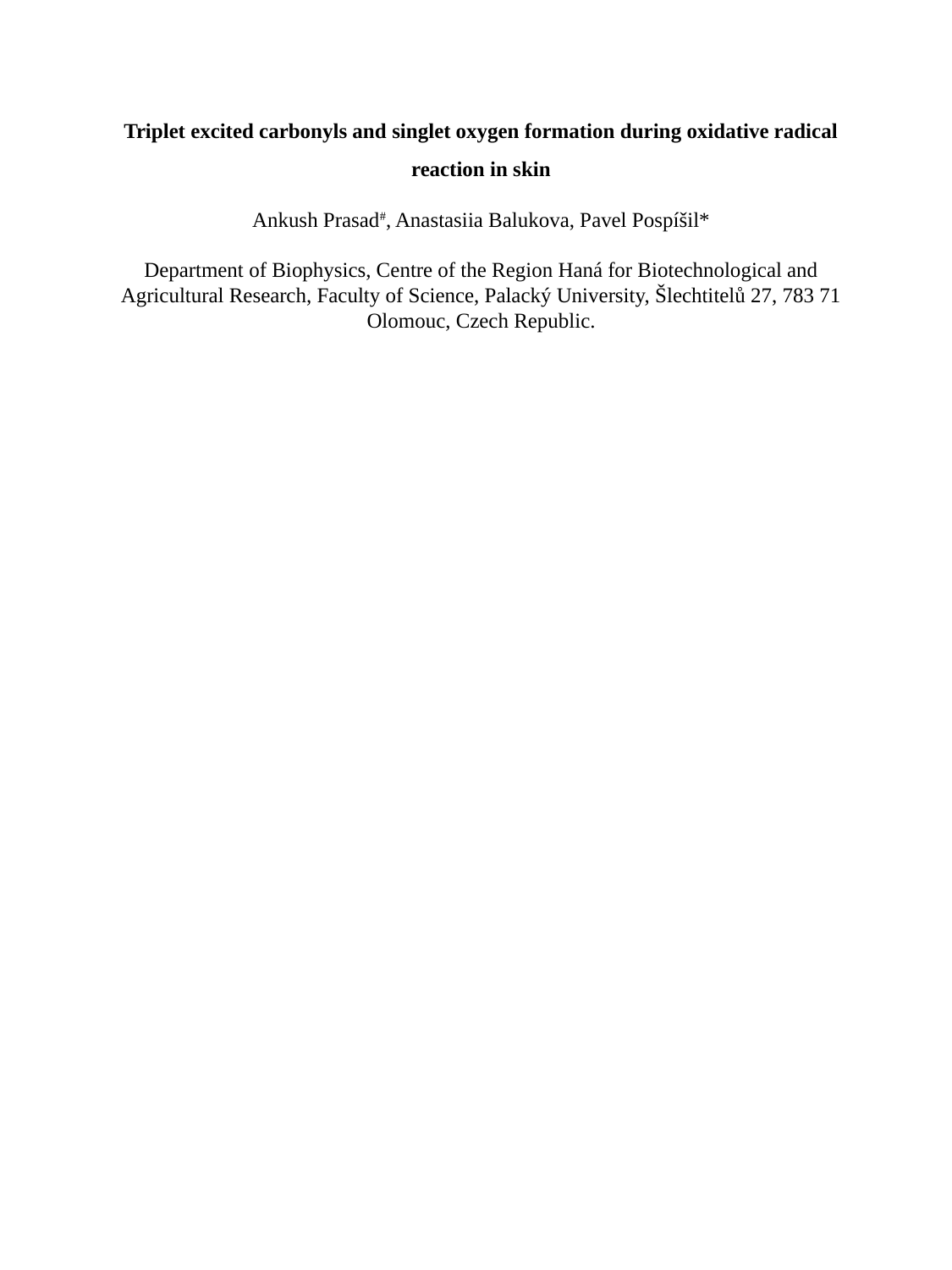

Triplet excited carbonyls and singlet oxygen formation during oxidative radical reaction in skin
Ankush Prasad#, Anastasiia Balukova, Pavel Pospíšil*
Department of Biophysics, Centre of the Region Haná for Biotechnological and Agricultural Research, Faculty of Science, Palacký University, Šlechtitelů 27, 783 71 Olomouc, Czech Republic.

## Slide 2
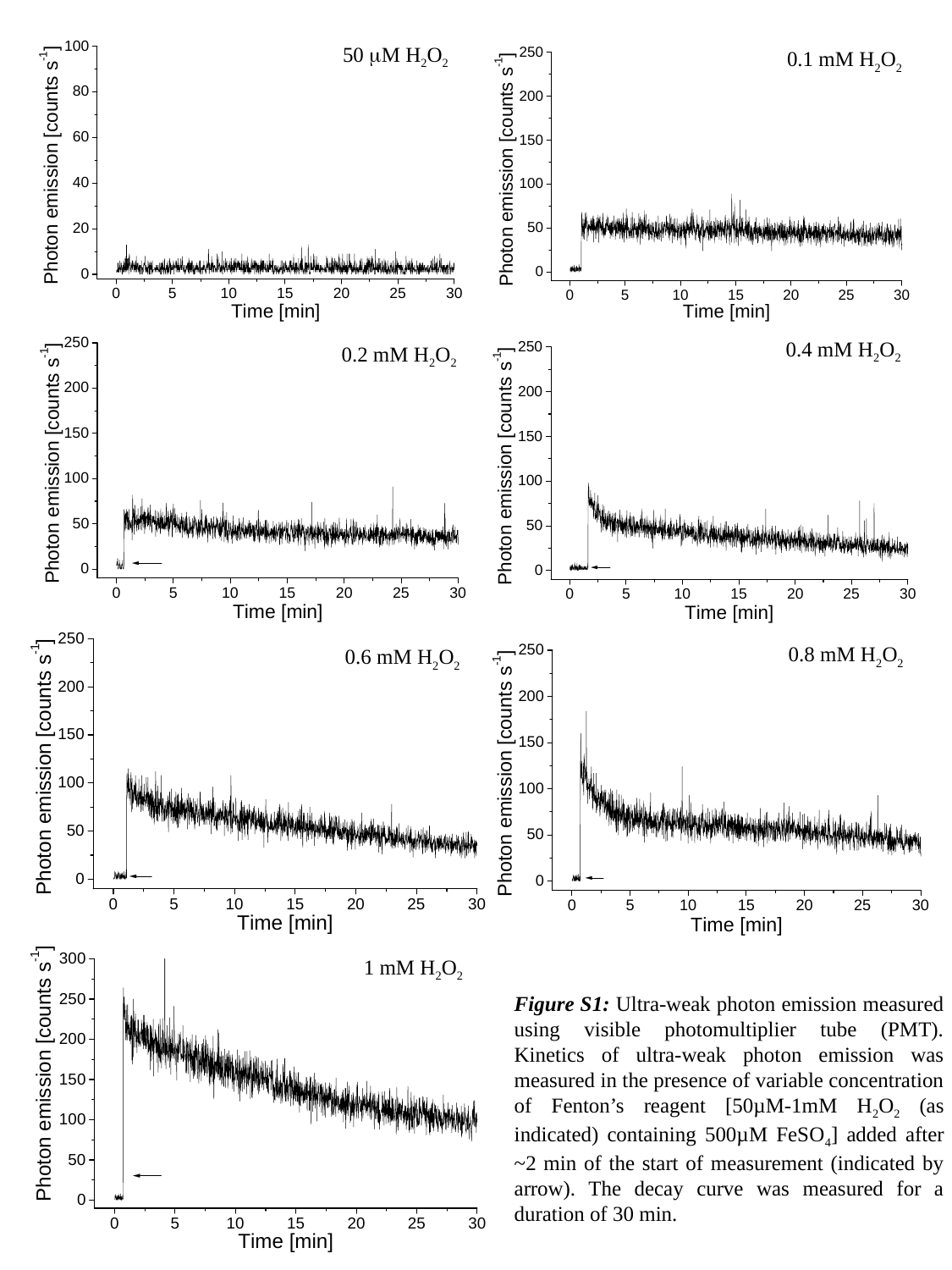

50 mM H2O2
0.1 mM H2O2
0.4 mM H2O2
0.2 mM H2O2
0.8 mM H2O2
0.6 mM H2O2
1 mM H2O2
Figure S1: Ultra-weak photon emission measured using visible photomultiplier tube (PMT). Kinetics of ultra-weak photon emission was measured in the presence of variable concentration of Fenton’s reagent [50µM-1mM H2O2 (as indicated) containing 500µM FeSO4] added after ~2 min of the start of measurement (indicated by arrow). The decay curve was measured for a duration of 30 min.

## Slide 3
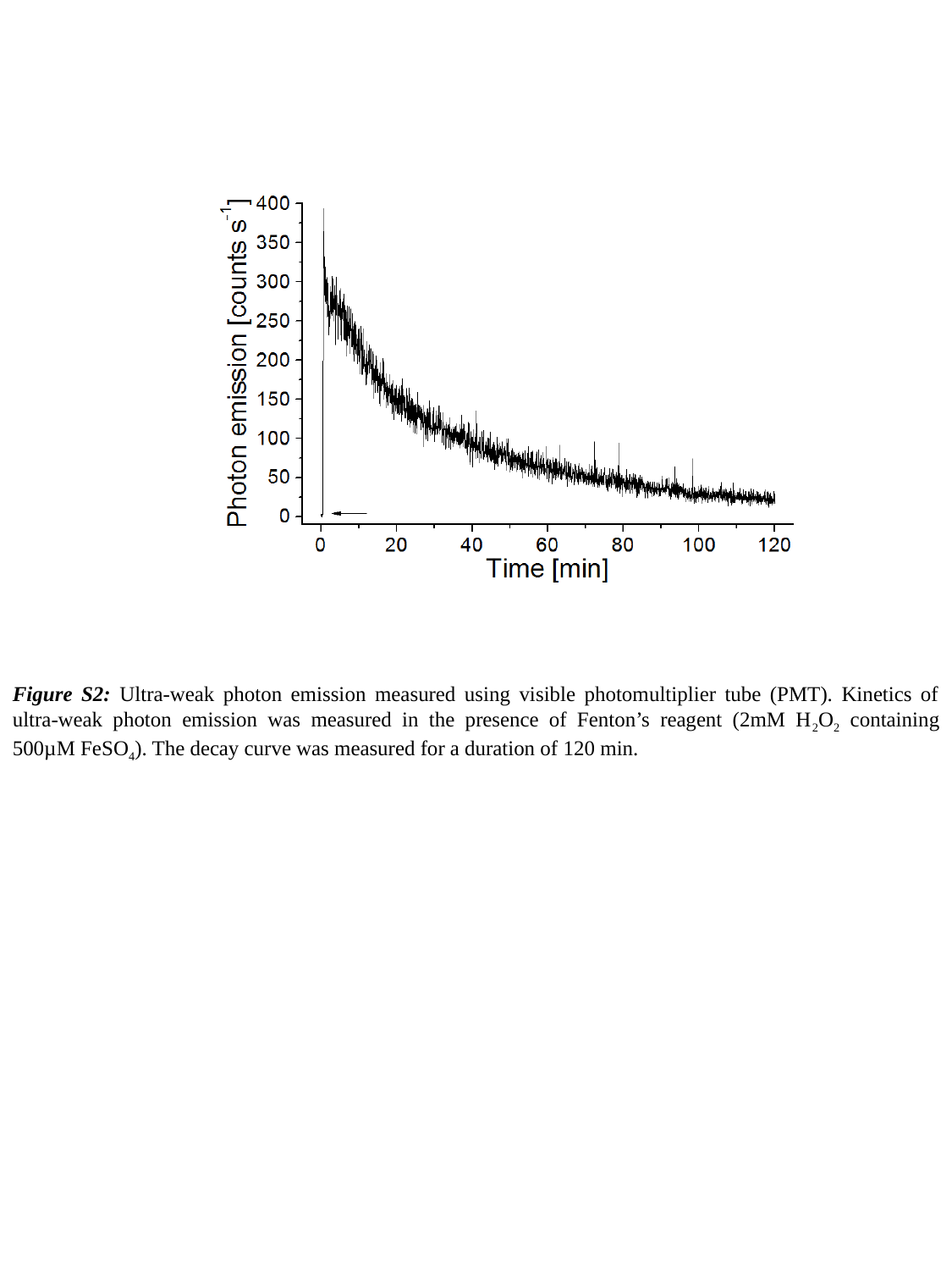

Figure S2: Ultra-weak photon emission measured using visible photomultiplier tube (PMT). Kinetics of ultra-weak photon emission was measured in the presence of Fenton’s reagent (2mM H2O2 containing 500µM FeSO4). The decay curve was measured for a duration of 120 min.

## Slide 4
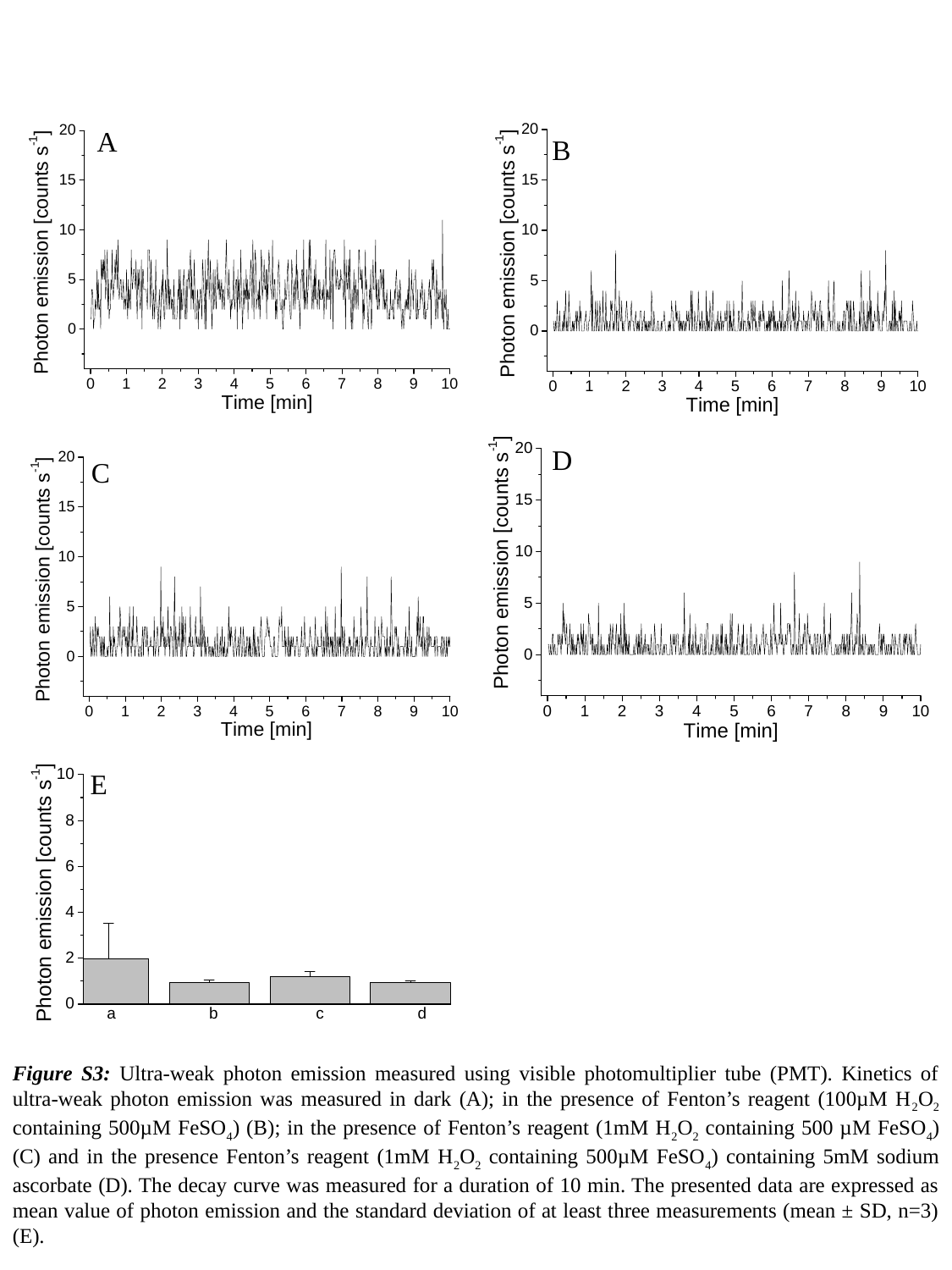

A
B
D
C
E
Figure S3: Ultra-weak photon emission measured using visible photomultiplier tube (PMT). Kinetics of ultra-weak photon emission was measured in dark (A); in the presence of Fenton’s reagent (100µM H2O2 containing 500µM FeSO4) (B); in the presence of Fenton’s reagent (1mM H2O2 containing 500 µM FeSO4) (C) and in the presence Fenton’s reagent (1mM H2O2 containing 500µM FeSO4) containing 5mM sodium ascorbate (D). The decay curve was measured for a duration of 10 min. The presented data are expressed as mean value of photon emission and the standard deviation of at least three measurements (mean ± SD, n=3) (E).
